# Pushing the Envelope: The Mysterious Journey Through the Bacterial Secretory Machinery, and Beyond

**DOI:** 10.3389/fmicb.2021.782900

**Published:** 2021-11-30

**Authors:** Luca A. Troman, Ian Collinson

**Affiliations:** School of Biochemistry, University of Bristol, Bristol, United Kingdom

**Keywords:** protein secretion, bacterial envelope biogenesis, SecY translocon, BAM, periplasm, SecDF

## Abstract

Gram-negative bacteria are contained by an envelope composed of inner and outer-membranes with the peptidoglycan (PG) layer between them. Protein translocation across the inner membrane for secretion, or insertion into the inner membrane is primarily conducted using the highly conserved, hourglass-shaped channel, SecYEG: the core-complex of the Sec translocon. This transport process is facilitated by interactions with ancillary subcomplex SecDF-YajC (secretion) and YidC (insertion) forming the holo-translocon (HTL). This review recaps the transport process across the inner-membrane and then further explores how delivery and folding into the periplasm or outer-membrane is achieved. It seems very unlikely that proteins are jettisoned into the periplasm and left to their own devices. Indeed, chaperones such as SurA, Skp, DegP are known to play a part in protein folding, quality control and, if necessary degradation. YfgM and PpiD, by their association at the periplasmic surface of the Sec machinery, most probably are also involved in some way. Yet, it is not entirely clear how outer-membrane proteins are smuggled past the proteases and across the PG to the barrel-assembly machinery (BAM) and their final destination. Moreover, how can this be achieved, as is thought, without the input of energy? Recently, we proposed that the Sec and BAM translocons interact with one another, and most likely other factors, to provide a conduit to the periplasm and the outer-membrane. As it happens, numerous other specialized proteins secretion systems also form trans-envelope structures for this very purpose. The direct interaction between components across the envelope raises the prospect of energy coupling from the inner membrane for active transport to the outer-membrane. Indeed, this kind of long-range energy coupling through large inter-membrane assemblies occurs for small molecule import (e.g., nutrient import by the Ton complex) and export (e.g., drug efflux by the AcrAB-TolC complex). This review will consider this hypothetical prospect in the context of outer-membrane protein biogenesis.

## Summary

The Gram-negative bacterial cell envelope is typically composed of inner and outer-membranes with the periplasm sandwiched in between. The inner membrane is the first membrane in contact with the cytoplasm and is therefore central to energy conservation, lipid biosynthesis, protein secretion and transport. The inner membrane itself mostly consists of phospholipids and hydrophobic α-helical proteins. In particular, the inner membrane houses the conserved ubiquitous Sec secretion machinery providing a sophisticated transport pathway for unfolded protein substrates across or into the membrane. Importantly, the inner membrane is necessary for the proton motive force (PMF), which consists of an electrical (ΔΨ) and chemical (ΔpH) gradient across the inner membrane created by proton transport into the periplasm. Energy conserved in this process can be converted into kinetic movement of several different membrane proteins to assist in transport across the membrane, or to drive energetically unfavorable reactions such as the phosphorylation of adenosine di-phosphate (ADP) to adenosine tri-phosphate (ATP) by ATP synthase. In addition, the PMF powers the rapid rotation of the flagella for movement of the bacterium itself.

The periplasm is a crowded aqueous compartment containing chaperones and proteases for protein quality control and folding, enzymes involved in sugar and amino acid transport and chemotaxis and nucleases preventing uptake of foreign DNA (Weski and Ehrmann, 2012). In addition the periplasm contains the peptidoglycan layer, a structural polymer tethered to the outer-membrane. The role of the peptidoglycan layer is to provide mechanical strength to withstand the cytoplasmic turgor, and maintain the cellular shape. As the name suggests, the peptidoglycan layer is a matrix of glycan strands cross-linked by short peptides; any inhibition to biosynthesis of the peptidoglycans through either mutation or antibiotic inhibition will result in cell lysis (Dezélée and Bricas, 1970).

The primary function of the outer-membrane is to protect the bacteria from the harsh surrounding environment. Additionally, resident membrane proteins are involved in solute and protein translocation and signal transduction. Like the inner membrane, the internal leaflet of is made up of phospholipids, but in bacteria with an outer-membrane the external leaflet of the bilayer is exclusively glycolipids. Unlike the inner membrane, proteins within this outer-membrane environment primarily consist of β-barrels.

## Crossing the Inner Membrane

Multiple specialized secretion systems have evolved for flagella assembly and for pathogenic contact and protein delivery to host cells (for review see Green and Mecsas, 2016). Many of these systems deploy trans-envelope assemblies to enable uninterrupted protein passage through the periplasm. Most extra-cytosolic proteins, however, are transported through the Sec secretion system (Orfanoudaki and Economou, 2014). This general secretion system spans only the inner membrane and allows transport of unfolded polypeptides destined for the inner membrane, periplasm, or outer-membrane. Unlike the specialized systems, this is highly conserved and abundant through bacteria, as well as archaea and eukaryotes.

The majority of the components for the Sec secretion system were identified through genetic screening experiments in the 1980s (for review see Bieker and Silhavy, 1990). Mutations resulting in a secretion deficiency were dubbed sec alleles, and corresponded to genes for SecY, SecE, SecD, SecF, SecA, and SecB (Emr et al., 1981; Oliver and Beckwith, 1981; Kumamoto and Beckwith, 1983; Riggs et al., 1988). Suppressor mutations causing hyperactive secretion characterized by a lack of sensitivity to the signal sequence mutations—*prl* alleles—corresponded to SecY (*prlA*), SecE (*prlG*), SecG (*prlH*), and SecA (*prlD*) (Oliver and Beckwith, 1981; Schatz and Beckwith, 1990; Schatz et al., 1991; Bost and Belin, 1997).

The first high-resolution structure for the Sec translocon was determined for SecYEG from *Methanococcus jannaschii* (Van Den Berg et al., 2004) allowing the first visualization of a protein conducting channel, in this case at the core of the Sec machinery. Ten transmembrane helices of SecY form two rigid bodies around a central channel with a lateral gate, allowing protein movement across the membrane as well as lateral movement into the phospholipid membrane itself (Van Den Berg et al., 2004; Egea and Stroud, 2010). For transport through the SecY channel the pre-secretory protein must be unfolded (Arkowitz et al., 1993) and this requires a carefully choreographed network of interactions between proteins within the cytoplasm, periplasm and inner membrane to enable efficient translocation of such a broad range of substrates.

The assignment of the pathway for transport across or into the inner-membrane is specified by translation of a short hydrophobic signal sequence on the N-terminus of the secretory/membrane protein (Blobel and Sabatini, 1971; Milstein et al., 1972; Blobel and Dobberstein, 1975). A bespoke toolkit of secretory machinery will ultimately determine the means of translocation of unfolded proteins through and into the membrane. These can loosely be separated as two different pathways.

• *Co-translational translocation:* a translating ribosome nascent chain complex binds to the SecYEG membrane channel usually resulting in membrane insertion.

• *Post-translational/SecA-dependent translocation:* a fully synthesized pre-protein from the cytoplasm is driven through the SecYEG membrane channel using molecular motor SecA, this usually results in membrane translocation into the periplasm.

The hydrophobic signal sequence, preceded by a positive charge at the N-terminus, makes up a recognition site for a range of factors to bind and target the substrate toward the inner membrane (Von Heijne, 1981). Generally, it is the hydrophobicity which then determines whether the pre-protein is transported after or during translation, with more hydrophobic sequences favoring co-translational translocation and less hydrophobic favoring post-translational translocation (Lee and Bernstein, 2001).

**SecA-dependent post-translocation transport** across the inner membrane, driven by ATP and the PMF, has been covered in a previous *Frontiers in Microbiology* review (Collinson, 2019). Here we are concerned about what happens after the translocation process. An important aspect of this consideration is the composition of the protein-channel complex. The core-SecYEG complex is necessary and sufficient for protein transport (Brundage et al., 1990), but other factors are known to associate in order to facilitate the reaction. The key point here is that the core complex does not have any appreciable domains at the periplasmic surface. So, on its own SecYEG does not seem to be equipped to effectively manage the release of emerging unfolded polypeptides to secure safe passage to the periplasm or outer-membrane. However, other factors known to associate with the core-complex (the subcomplex SecDF, YidC, YajC, PpiD, YfgM) (Götzke et al., 2014; Schulze et al., 2014; Jauss et al., 2019), possess such exterior domains large enough to be involved in the onward traffic of protein.

## The Ancillary Sub-Complex SecDF

Genes encoding both SecD and SecF are co-transcribed within a single operon in *Escherichia coli* (*E. coli*) indicative of the propensity for SecDF complex formation (Gardel et al., 1990). SecDF is not essential in bacteria, but SecDF null strains have heavily impaired translocation leaving them barely viable (Gardel et al., 1990; Pogliano and Beckwith, 1994), whilst the over-expression of SecDF stimulates translocation (Pogliano and Beckwith, 1994). However, the precise mechanism of how SecDF assists translocation is unclear. Early co-immuno precipitation (co-IP) experiments using antibodies for SecG identified SecDF as an interactor with SecYEG as part of a larger complex containing YajC and another protein now identified as YidC (Duong and Wickner, 1997a). This complex was shown to prevent the backsliding of proOmpA translocation intermediates during translocation through the SecYEG channel (Duong and Wickner, 1997b). YajC, SecD and SecF also form a heterotrimer independently of other translocation machinery (Pogliano and Beckwith, 1994).

**FIGURE 1:**
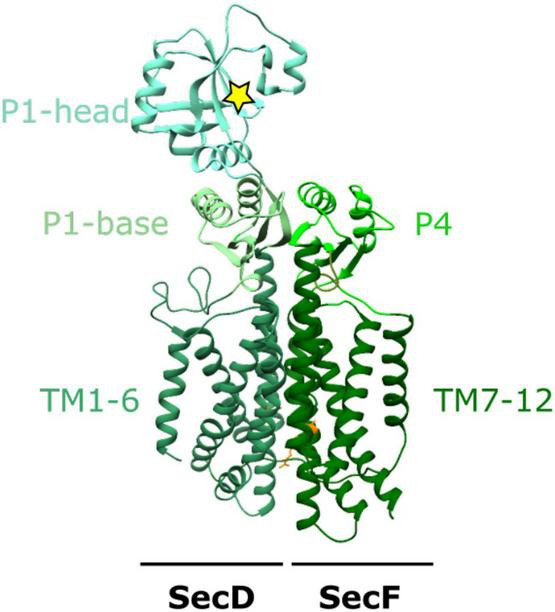
The structure of SecDF. X-ray crystal structure of SecDF in I- form conformation from *Deinococcus radiodurans* R1 PDB:5XANb (Furukawa et al., 2017). TM1-6 from SecD and TM7-12 from SecF make up the 12 TM helices characteristic of the RND superfamily. The periplasmic domains: P1-base and -head in SecD and P4 in SecF, interact to form a domain which moves in response to proton influx through a proton wire (including SecD_*D*519_ and SecF_*R*247_; orange) within the membrane domain. The yellow star depicts a hydrophobic pocket within the P1-head of SecD.

SecDF is a member of the resistance-nodulation-division (RND) superfamily, containing 12 TM helices, split between SecD and SecF each with 6 TM helices shown in Figure 1. SecDF has low homology with other members of the family with the largest differences manifesting in the large and structurally flexible periplasmic domains (P1-head and base and P4), the movement of which is coupled to the movement of protons down their electrostatic and chemical gradient (PMF).

There are multiple high-resolution crystallographic structures solved for the SecDF complex (Tsukazaki et al., 2011; Furukawa et al., 2017, 2018). Each of these differ in both the transmembrane and periplasmic domains, with the most drastic conformational changes observed in the periplasmic domains. The P1 “head” domain of SecD undergoes a 100 degree rotation to move between “F” and “I” forms resulting from a series of smaller conformational changes within the TM region of the complex (Tsukazaki et al., 2011; Furukawa et al., 2017). Cysteine crosslinks “locking” SecDF in this extended I form for crystallographic studies reveal the TM region can accommodate a central channel containing water molecules (Furukawa et al., 2017). This allows the movement of protons across the membrane via a proton wire formed by the water molecules and conserved arginine and aspartate residues. In particular, the deprotonation and reprotonation of a central conserved aspartate residue (D365 in *T. thermophilia*, D519 in *E. coli*) is critical for proton transport. Importantly, this channel is not always present in SecDF but the presence of the channel is likely to be coupled to a series of conformational changes important to the function of SecDF (Furukawa et al., 2017).

A final structural study captured a further more compact state where β-sheets in P1-base and P4 domains rearrange into a single β-barrel within the periplasm; this has been named the “Super-F” form (Furukawa et al., 2018). These movements depend on the interaction between two highly conserved residues at the center of the proton channel in SecDF (in *E. coli* D519 in SecD and R247 in SecF) (Furukawa et al., 2018). Mutagenesis of either of these central residues prevents SecDF reaching this super-F form and “locks” the SecDF into the I form (Furukawa et al., 2018).

The structural studies suggest that proton transport through the membrane is coupled to large conformational changes in the periplasm, which could indeed be involved in protein handling—perhaps for the emergence of polypeptides from the protein-channel through SecY and subsequent onward passage into the periplasm, or further afield toward the outer-membrane.

## Membrane Protein Insertion and YidC

Another associate of the core SecYEG complex is the membrane protein YidC—part of the YidC/Oxa1/Alb3 family of membrane “insertases” found across all domains of life. Members of this family are able to act autonomously or cooperatively with other Sec components to facilitate membrane protein assembly. In *E. coli* YidC is thought to function alone for the insertion of small membrane proteins including the c-subunit of F_1_F_*o*_-ATP synthase F_*o*_c (Van Der Laan et al., 2004), homopentameric channel protein MscL (Facey et al., 2007), phage coat proteins M13 and Pf3 (Samuelson et al., 2001; Chen et al., 2002), and the tail-anchored membrane protein TssL (Aschtgen et al., 2012). However, it also forms a network of interactions with ribosomes, signal recognition particle (SRP), SecYEG and SecDF where it cooperates to play a much larger role in membrane insertion and membrane protein complex formation. Numerous membrane proteins have been shown to require both YidC and SecY for successful membrane insertion (Nagamori et al., 2004; Celebi et al., 2006; Zhu et al., 2013). Studies from this authors research group even suggest that the small proteins thought to depend only on YidC enter the membrane more efficiently when SecYEG is also present (in the holo-translocon; HTL—see below) (Komar et al., 2016).

**FIGURE 2:**
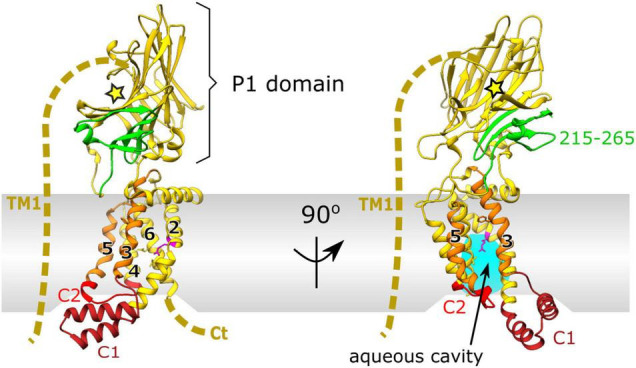
X-ray crystal structure of YidC from *E. coli* PDB:6AL2 (Tanaka et al., 2018). Cytoplasmic loop C1 (dark red) and flexible cytoplasmic C2 loop (red). The conserved 5 helical core of TM2-5 is shown in yellow with hydrophobic, greasy slide helices TM3 and 5 highlighted in orange. Periplasmic domain (P1) is shown in yellow, with the SecF interaction site in green (residues 215–265) and the substrate binding cleft depicted by a yellow star. The unknown structure of non-conserved TM1 and the C-terminus is depicted by a dashed yellow line. Conserved arginine residue (R366) on TM2 of the hydrophilic groove, important for insertion, within the aqueous cavity is shown in magenta.

Interestingly unlike SecY, members of the YidC/Oxa1/Alb3 family lack any form of channel and instead utilize a conserved helical bundle of only 5 TM helices and a conserved cytoplasmic coiled coil helix. This transmembrane part of YidC is sufficiently conserved across different lifeforms that expression of the family member Alb3 from chloroplasts, Oxa1 from mitochondria in *Saccharomyces cerevisiae*, or Alb4 from Arabidopsis can restore growth in YidC- depleted strains of *E. coli* (Jiang et al., 2002; Van Bloois et al., 2007; Benz et al., 2009). This 5 helical bundle is arranged with a “greasy slide” and a “hydrophilic groove” to interact with both the inner membrane and membrane protein substrate (Figure 2). Through a series of both hydrophilic and hydrophobic interactions the activation barrier for membrane protein insertion is reduced resulting in membrane protein insertion.

In addition to the known homology of YidC with Oxa1 and Alb3/4 within mitochondria and chloroplasts (for review see Hennon et al., 2015), recent studies have identified both sequence and structural homology between the prokaryotic YidC and some components of the secretory pathway within eukaryotes (Anghel et al., 2017; Bai et al., 2020; McDowell et al., 2020; Pleiner et al., 2020). Within the conserved endoplasmic reticulum membrane complex (EMC) in eukaryotes TM helices of different Emc components interact to form a YidC-like fold (Bai et al., 2020; Pleiner et al., 2020). Additionally Get1/2 in the guided entry of tail-anchored proteins (GET) pathway in *Saccharomyces cerevisiae*, and the homologous human WRB/CAML both contain hydrophilic grooves analogous to those in prokaryotic YidC (McDowell et al., 2020). This suggests that the hydrophilic groove within YidC is representative of an evolutionary conserved insertion mechanism for membrane protein substrates.

Unlike many other family members in mitochondria, or Gram-positive bacteria, the Gram-negative counterpart has a large and flexible periplasmic domain (P1; Figure 2). Removal of this periplasmic domain in *E. coli* has no effect on its membrane insertion capabilities (Jiang et al., 2003). It is therefore likely that the P1 domain of YidC has a distinct role in *E. coli* not observed elsewhere, e.g., in mitochondrial Oxa1 or Gram-positive SpoIIIJ which lack this domain. Through mutagenesis experiments, residues 215–265 of this region have been identified as the interaction site for SecF of the SecDF subcomplex (shown in green in Figures 1, 2; Xie et al., 2006) although the full function of this domain within the periplasm remains unclear.

The P1 domain has some structural similarities to galactose mutarotase, including a central cleft corresponding to a sugar binding pocket, but lacking the sugar binding motif (Oliver and Paetzel, 2008; Ravaud et al., 2008). In one crystallographic study a polyethelyene glycol (PEG) molecule from the crystallization buffer co-crystallized within the cleft forming extensive hydrophobic interactions with 22 different conserved residues (Ravaud et al., 2008). Notably, PEG molecules have been observed to occupy clefts that naturally bind elongated polymers such as polypeptides in chaperones, or long acyl chains in enzymes and typically form similar interaction networks as that observed within P1 cleft of YidC (Ravaud et al., 2008). Given the hydrophobic binding capabilities of the cleft, the authors of the study suggest a few potential roles for the P1 domain *in vivo*; (*i*) it could interact with the peptidoglycan layer, (*ii*) potentially it could interact with an elongated peptide chain from an interacting protein in the periplasm, or alternatively (*iii*) it could have a role as a molecular chaperone through interaction with an unfolded polypeptide in the periplasm. Further structural studies have now revealed that this cleft is oriented away from the membrane (Kumazaki et al., 2014), hence interactions with molecules or interacting protein in the periplasm are more likely than with unfolded polypeptide.

## The Interaction of YidC With SecYEG and SecDF

Interactions between YidC and the Sec translocon were first discovered through co-purification with SecYEG (Scotti et al., 2000). These co-purification experiments showed that the over-production of either SecYEG or the SecDF-YajC subcomplex caused an increase in the levels of YidC, implicating a cooperative role in SecDF and SecYEG dependent translocation (Scotti et al., 2000). Subsequent studies revealed the requirement for both SecYEG and YidC in insertion of larger polytopic membrane proteins (Nagamori et al., 2004; Celebi et al., 2006; Zhu et al., 2013). It is now known that YidC forms intimate contacts with the lateral gate and pore ring residues within the channel of SecY (Sachelaru et al., 2013, 2017). This positions it well to receive and facilitate the transition of trans-membrane helices from the channel, *via* the “greasy slide” to the bilayer. Additionally more recent *in vitro* and *in vivo* crosslinking experiments identified the auxiliary TM1 of *E. coli* YidC as a major contact site for interaction with both SecG and SecY (Petriman et al., 2018; Figure 2). These also implicate the C1 loop and C2 cytoplasmic domains as major interaction sites with SecY confirming that the hydrophilic cavity of YidC faces the lateral gate of SecY (Petriman et al., 2018).

## The Holo-Translocon Complex

The interactions between SecYEG, SecDF-YajC and YidC led to the concept of a large holo-complex: whereby efficient protein secretion and membrane insertion, through the core-complex, is facilitated by associated ancillary factors, respectively SecDF and YidC. This was first recognized through co-immuno-precipitation of SecYEG and SecDF-YajC (Duong and Wickner, 1997a). Much later it was shown that a complex containing SecYEG, SecDF-YajC and YidC could be extracted from native membranes; achieved by SMALPS (styrene maleic anhydride lipid particles) extraction, which retains the lipid bilayer around solubilized membrane proteins (Komar et al., 2016). Balanced over-production of SecYEG, SecDF-YajC and YidC (Bieniossek et al., 2009), enabled the isolation and characterization of the this “holo-translocon” (HTL) (Schulze et al., 2014). The HTL was indeed capable of post-translational ATP and PMF driven transport through SecYEG by SecA, as well as co-translational membrane protein insertion (Schulze et al., 2014).

**FIGURE 3:**
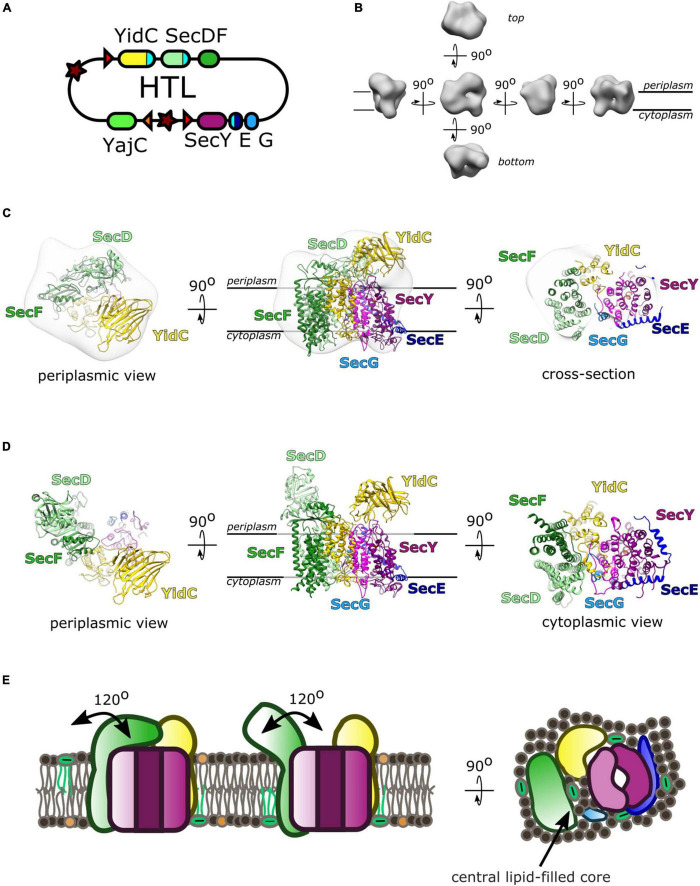
The Holo-translocon of *E. coli*. **(A)** Plasmid system for expression of HTL (Bieniossek et al., 2009). **(B)** 14 Å resolution electron density of HTL in amphipols (EMD:3506; Botte et al., 2016). **(C)** Computational fitting of individual components of HTL to this density (PDB:5MG3 Botte et al., 2016). SecDF (green; SecD P1 domain, light green), YidC (yellow), SecYEG (magenta, dark blue, light blue). **(D)** A suggested alternate conformation of this complex where SecDF periplasmic domain is rotated into the *I*-form (Martin et al., 2019). **(E)** Schematic of HTL depicting SecDF rotation and the central lipid cavity.

A low resolution (14 Å) cryo-electron microscopy (cryo-EM) structure allowed the determination of the arrangement of the individual components within the HTL (Botte et al., 2016). This placement used prior knowledge of component interactions, and computational fitting within the density (Figure 3; Botte et al., 2016). Efforts to improve the structure, to gain more insights of the interactions between SecYEG and the ancillary factors, have been thwarted by the inherent flexibility (necessary for function) of the assembly.

Interestingly, Martini course-grained (CG) molecular dynamic simulations and small-angle neutron scattering (SANS) experiments revealed a central lipid pool (∼8–29 phospholipids) between SecYEG, YidC and SecDF-YajC (Botte et al., 2016; Martin et al., 2019). Further analysis of the data suggests that both the protein and lipid components of the HTL are highly dynamic. In the former case, this corresponds to movement of SecD between I- and F-forms (Figure 3D; Furukawa et al., 2017), and in the latter lipids exchanging between the central pool and the surrounding bilayer (Martin et al., 2019).

This lipid core could create a suitable environment for membrane protein assembly, protected from surrounding membrane proteins which could cause aggregation or proteolysis (Figure 3E). This concept is reminiscent of the mechanism deployed by the chaperonin GroEL, which provides an aqueous cavity to promote efficient folding of globular proteins (Ranson et al., 1997; Xu et al., 1997). The likely dynamic nature of this pool would be suited for the accommodation and folding of differently sized membrane proteins on their way to the membrane.

## Additional Associates of the Sec Machinery

There is a very strong possibility that other factors associate with the translocon. This could be to confer new functionalities, or to regulate and refine protein secretion, insertion and quality-control activities. Additional factors will also be needed for the management of the onward passage of proteins for degradation or for their safe passage through the periplasm.

Membrane tethered periplasmic chaperone PpiD with its partner protein YfgM has been shown to interact with SecY (Götzke et al., 2014; Fürst et al., 2018; Jauss et al., 2019). The interaction sites appear to overlap with the those of YidC at the lateral gate. Thus, these factors might associate to fine tune the properties of the core-SecYEG complex according to the transiting client: YidC for insertion and PpiD/YfgM for periplasmic processing downstream of secretion. These and other complex interaction networks must operate to ensure the versatility of the Sec translocon enabling the effective delivery of a vast quantity and diversity of the proteins across the bacterial envelope.

## Crossing the Periplasm

**FIGURE 4:**
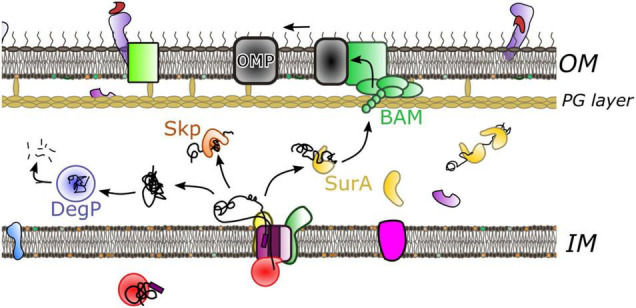
Crossing the periplasm. Following protein translocation through the inner membrane multiple pathways operate during onward passage. The predominant chaperone SurA (yellow) holds the protein in an unfolded state prior to delivery to the outer-membrane through interaction with the BAM complex. Alternatively the chaperone Skp (orange) has aggregate recovery activity. If the peptide misfolds or aggregates DegP protease (blue) will degrade the substrate.

Proteins exiting through the SecY-channel need to be sorted for delivery and folding into the periplasm or directed to the outer-membrane (Figure 4). Irrespective of their final destination the newly transported polypeptide must be maintained in a non-aggregated and non-folded state, or otherwise degraded if things go wrong—e.g., misfolding, channel-blocking, aggregation. In order to achieve this in the very challenging environment of the periplasm (Pedebos et al., 2021), a range of periplasmic chaperones have evolved, in the absence of any apparent energy source. SurA and Skp are thought to be the most prominent of these quality control factors, working in conjunction with DegP periplasmic protease (Rizzitello et al., 2001; Sklar et al., 2007; Li et al., 2018). These parallel and redundant pathways provide a robust means to navigate the dense proteinaceous environment. SurA has been shown, by chemical cross-linking, to interact with BamA—the major subunit of the BAM complex, suggestive of a role in the delivery to the outer-membrane (Sklar et al., 2007). Although, how the chaperoned proteins travel beyond the PG layer is unclear.

**SurA** plays a major role in OMP biogenesis; its deletion leads to OMP assembly defects and changes in outer-membrane composition. This in turn induces the stress response and causes increased susceptibility to external factors such as antibiotics (Lazar and Kolter, 1996; Rouvière and Gross, 1996; Behrens et al., 2001; Justice et al., 2005; Vertommen et al., 2009; Klein et al., 2019). The phenotype is severe, but falls short of lethality—null strains are viable due to redundancy with DegP and Skp (Sklar et al., 2007).

**FIGURE 5:**
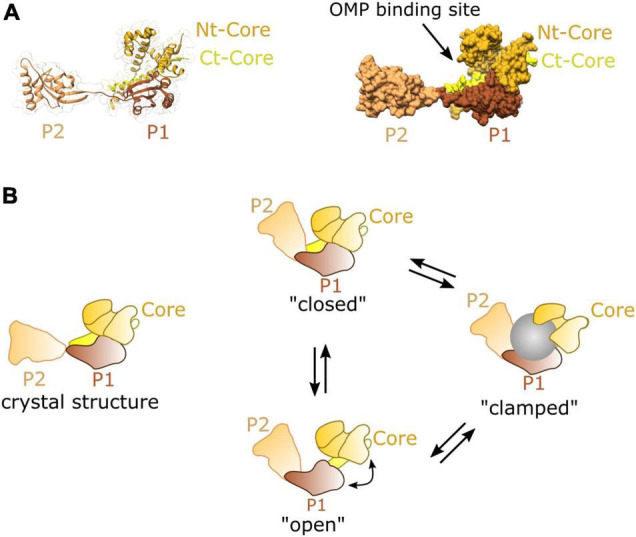
Structure and dynamics of SurA. **(A)** Cartoon (left) and surface (right) representations of the crystal structure of SurA from *E. coli* PDB:1M5Y (Bitto and McKay, 2002). This contains a core domain consisting of the N and C terminal sequences, and P1 and P2 parvulin-like PPIase domains. **(B)** A model of dynamic movements between conformations distinct from the crystal structure as revealed from single molecule FRET experiments, adapted from Calabrese et al. (2020).

The structure of SurA reveals a core domain split into N- and C- terminal regions, and two parvulin-like peptidylproyl isomerase (PPIase) domains (P1 and P2) (Bitto and McKay, 2002). SurA has a preference for binding aromatic sequences with Ar-X-Ar motif (Bitto and McKay, 2002; Hennecke et al., 2005; Xu et al., 2007), a motif commonly found in C-terminal regions of many OMPs (Behrens-Kneip, 2010; Merdanovic et al., 2011). The site at which these peptide motifs interact with the SurA chaperone has not been identified, but the crystallographic structure reveals an extended crevice within the core of domain which could be involved in the binding and release of the peptide substrate during folding (Figure 5A; Bitto and McKay, 2002). Single-molecule FRET experiments indicate that two SurA molecules can bind a single OmpC substrate, but that the stoichiometry may differ depending on the size of the substrate (Li et al., 2018).

Recent studies using FRET and chemical cross-linking, revealed that in solution SurA adopts multiple conformations all of which substantially differ from the crystal structure (Figure 5B; Calabrese et al., 2020). Conformational analysis of this data suggests that the three domains of SurA form a cradle around OMP clients protecting them from aggregation (Calabrese et al., 2020). This structural plasticity may be an essential feature of SurA activity, as it is for many other chaperones (Burmann and Hiller, 2015; Suss and Reichmann, 2015). Importantly, the chaperone function of SurA seems to span from early to late stages of the journey forming interactions at both SecYEG (Ureta et al., 2007) and the BAM complex (Hennecke et al., 2005; Sklar et al., 2007; Gunasinghe et al., 2018).

**Skp**, like SurA, binds a broad range of substrates, sequestering them from the dense and protein-packed periplasmic space (Qu et al., 2007; Wu et al., 2011). Whilst neither SurA or Skp are required for cell viability, cells lacking SurA must contain both Skp and protease DegP, which thereby compensate for the loss of SurA activity (Rizzitello et al., 2001; Sklar et al., 2007).

**FIGURE 6:**
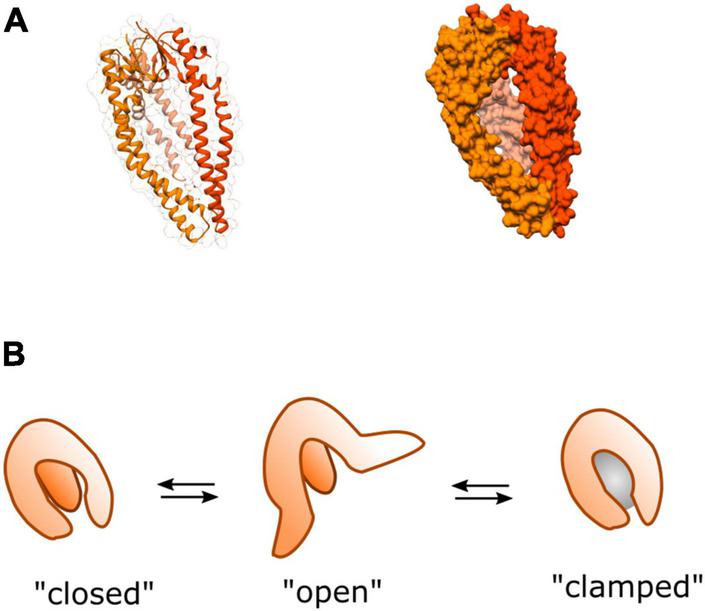
Structure and dynamics of Skp. **(A)** Cartoon (left) and surface (right) representations of the crystal structure of homo-trimeric periplasmic chaperone Skp of *E. coli* PDB:1SG2 (Korndörfer et al., 2004). **(B)** A model of dynamic movements between conformations adapted from Holdbrook et al. (2017). The three tentacular arms function to clamp around substrates of different sizes (represented by the gray sphere) binding them in the hydrophobic core to prevent aggregation, or rescue partially aggregated proteins.

Unlike SurA which recognizes specific hydrophobic sequences (Bitto and McKay, 2002; Hennecke et al., 2005; Xu et al., 2007), Skp forms a large jellyfish-like trimeric structure (Korndörfer et al., 2004; Walton and Sousa, 2004), interacting with protein substrates through non-specific hydrophobic and electrostatic interactions within a large hydrophobic cavity (Jarchow et al., 2008; Walton et al., 2009; Burmann et al., 2013; Callon et al., 2014; Figure 6). For a number of substrates the stoichiometry of Skp to substrate is 1:1 where the chaperone “swallows” the full substrate into the hydrophobic cavity (Bulieris et al., 2003; Qu et al., 2007; Lyu et al., 2012; Burmann et al., 2013) although large substrates can be accommodated by two Skp chaperones if it is too large for just one (Schiffrin et al., 2016). Like SurA, flexibility of Skp is thought to be integral to its function, where it operates as a spring-loaded clamp to wrap its tentacular arms around a range of different sized complexes into its hydrophobic core (Holdbrook et al., 2017).

In spite of the functional overlap with SurA, Skp has been shown to possess distinctive characteristics. In contrast to SurA, Skp has been shown to assist with folding of OMPs into the lipid bilayer *in vitro* (Bulieris et al., 2003; Patel et al., 2009; McMorran et al., 2013). Moreover, it has also been shown to be able to recover aggregated OMPs from the periplasm (Li et al., 2018). This activity could become especially important under cellular stress conditions where expression levels of several OMPs are up-regulated causing increased potential for aggregation. This is consistent with the finding that under stress conditions the expression of Skp is upregulated (Sklar et al., 2007), perhaps as a strategy to help prevent OMP aggregation during periods of high demand for outer-membrane biogenesis—e.g., rapid cell growth.

Like SurA, Skp has also been shown to interact with OMP during early stages of the translocation process (Harms et al., 2001). Although, as of yet, no interactions with the Sec or BAM machineries have been demonstrated (Sklar et al., 2007). This is consistent with the suggestion that the role of Skp is more prominent in OMP recovery and that SurA is the more important for OMP transport, although further clarification of their roles within the periplasm is required.

## Holo-Translocon and Barrel-Assembly Machinery: Inter-Membrane Association

**FIGURE 7:**
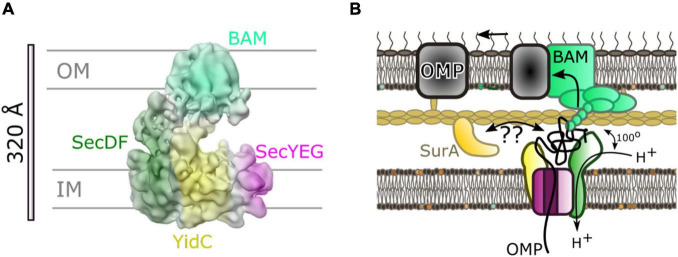
Inter-membrane association of HTL and BAM. **(A)** 14 Å cryo-EM density from Alvira et al. (2020) colored to show locations of BAM (light green) and components of HTL: SecDF (dark green), SecYEG (magenta) and YidC (yellow). **(B)** Cartoon of model for translocation through the HTL-BAM inter-membrane complex adapted from Alvira et al. (2020). Movement of periplasmic domains of SecDF (dark green) has a role in energy transduction of the PMF to the BAM complex for OMP maturation. The precise nature of the involvement of periplasmic chaperones is unknown; SurA is known to interact with OMPs soon after emergence from SecY and also with BAM on the other side of the envelope.

Even with the assistance of chaperones, it seems improbable that the OMP-chaperone complex can be reliant on diffusion alone for efficient transport through the periplasm. Photo-crosslinking studies (Wang et al., 2016), and more recently biochemical and low-resolution cryo-EM studies (Alvira et al., 2020) have implicated a direct connection between secretory components within the inner and outer-membranes. In particular a low-resolution cryo-EM reveals the interaction between the Sec and BAM translocons (Alvira et al., 2020; Figure 7). The assignment of different parts of the density to given complexes was inferred through difference negative stain electron microscopy of multiple subcomplexes with missing components, cross-linking and mass spectrometry. This revealed interactions between both periplasmic domains of HTL (on YidC and SecDF) and the periplasmic domains of BAM.

Mutations in the genes encoding SecDF causing amino acid substitution of an aspartate for an asparagine (approximating a deprotonated neutral aspartate), allow production of the SecDF variant (SecD_*D*519N_F), with a dysfunctional proton wire in the transmembrane portion of the complex. This version is known to bring about large changes in the conformation of the periplasmic domain P1 of SecDF (see above) (Tsukazaki et al., 2011; Furukawa et al., 2017, 2018). On this basis it is reasonably safe to assume that proton passage through SecD will promote conformational switching back and forth between the 2 states (perhaps similar to the *I-* and *F-*states) as aspartate-519 would alternate between the protonated and deprotonated forms. The analysis of this variant allowed the evaluation of the role of the proton coupled mobility of SecD in the context of its association with BAM, and subsequent OMP maturation. The results found that whilst the interaction with BAM components were unaffected, OMP maturation was considerably inhibited when compared to the wild-type strains, similar to the effect of a SecDF depletion (Alvira et al., 2020).

Previous publications have shown that SecYEG and SecA alone are sufficient for the PMF- and ATP-driven transport through the inner membrane, demonstrating that the SecDF proton driven conformational changes are not required for transport through the inner membrane. It follows that effects of the PMF conferred by SecDF must be important for the down-stream events critical to OMP maturation. Potentially, this could be through a dynamic interaction with the BAM complex (Figure 7).

One compelling explanation linking PMF-dependent rearrangement of SecDF to OMP maturation, is that these movements act as a mechanism for energy transduction between the inner and outer-membrane; a concept consistent with observations of other systems. One example of long range energy transduction across the envelope is another RND transporter family member—the multi-drug efflux pump formed by the inter-membrane association of AcrAB (IM) and TolC (OM) (Du et al., 2014; Wang et al., 2017; Chen et al., 2021). Another is the Ton complex for nutrient import (Celia et al., 2016). Other striking parallels even contain homologs of BamA including: (*i*) double membrane spanning translocation and assembly modules (TAM) found in proteobacteria (Selkrig et al., 2012), and (*ii*) the TIC-TOC import machinery of chloroplasts (Chen et al., 2018) contain BamA homologs TamA and TOC75, respectively. The dynamically coupled double-membrane protein transport system suggested between HTL and BAM is therefore far from a novel concept; membrane coupling is a common occurrence within the envelopes of chloroplasts and bacteria, and probably mitochondria as well.

Interestingly, the low-resolution structure for the HTL-BAM complex contains a large periplasmic cavity, with ample room to accommodate periplasmic chaperones. SurA for example, is a known interactor of BamA and may well be stationed here. The obvious question remains about how these chaperones coordinate with HTL alone and with trans-envelope complex HTL-BAM complex in order to manage the lateral efflux of proteins either into the periplasm or the outer-membrane.

There is indeed a lot still to be learnt about the dynamic molecular mechanism and bioenergetics underlying the bacterial general secretory process. This remains true of the early stages of this process—the mechanism of ATP and PMF driven transport across the inner membrane by cytosolic SecA through SecYEG, and even more so for what needs to happen afterward. Knowledge of the early and, in particular, the later stages of this process is critical for our understanding of the fundamental process of bacterial envelope biogenesis. Moreover, future new insights will be critical in the development of strategies to compromise the cell wall toward the development of new anti-bacterials, or as supplements designed to potentiate cytotoxic agents which would otherwise be excluded from the cell.

## Author Contributions

LT and IC wrote the manuscript. Both authors contributed to the article and approved the submitted version.

## Conflict of Interest

The authors declare that the research was conducted in the absence of any commercial or financial relationships that could be construed as a potential conflict of interest.

## Publisher’s Note

All claims expressed in this article are solely those of the authors and do not necessarily represent those of their affiliated organizations, or those of the publisher, the editors and the reviewers. Any product that may be evaluated in this article, or claim that may be made by its manufacturer, is not guaranteed or endorsed by the publisher.
